# Prescribed Performance Attitude-Tracking Control for Rigid Satellite Under External Disturbance

**DOI:** 10.3390/s26072189

**Published:** 2026-04-01

**Authors:** Chunyu Zhang, Ting Wang, Min Wan, Tao Li

**Affiliations:** 1 College of Automation Engineering, Nanjing University of Aeronautics and Astronautics, Nanjing 211106, China; cyzhang@nuaa.edu.cn (C.Z.); wanmin@nuaa.edu.cn (M.W.); 2 College of Information Science and Technology, Nanjing Forestry University, Nanjing 210037, China; wangting10402@njfu.edu.cn

**Keywords:** satellite attitude system, tracking control, prescribed-time disturbance observer (PTDO), prescribed performance control (PPC)

## Abstract

This paper addresses the attitude control problem for a rigid satellite and proposes an anti-disturbance prescribed performance control (PPC) scheme, aiming to achieve accurate attitude tracking while guaranteeing tracking performance under external disturbances. First, a prescribed-time disturbance observer (PTDO) is developed to achieve the precise and rapid estimation of external disturbances within a prescribed time. Second, an appointed-time performance function (ATPF) is introduced, based on which an asymmetric performance boundary is constructed to ensure convergence within the appointed time. Subsequently, by enforcing the constructed sliding-mode error to satisfy prescribed performance constraint, the desired tracking performance of the satellite attitude system is achieved. Third, according to Lyapunov stability theory, it is proven that the disturbance estimation error and the unconstrained error in the attitude system are uniformly ultimately bounded, thereby enabling the achievement of the desired control performance. Finally, the effectiveness of the proposed control strategy is verified via numerical simulations.

## 1. Introduction

Owing to the rapid progress in aerospace technology, satellites have been increasingly utilized in missions including communication, deep-space exploration, and so on [1,2,3]. As a critical subsystem of spacecraft, accurate attitude control is essential to the successful accomplishment of satellite missions, since it has a direct impact on overall control performance [4,5]. Due to the pronounced nonlinear characteristics of satellite attitude control systems, as well as parameter uncertainties including mass and moments of inertia and complex space environmental disturbances, the design of high-performance controllers becomes significantly more challenging [6]. Meanwhile, practical applications typically require that both transient and steady-state performance specifications be satisfied. Therefore, the design of a high-performance controller for satellite attitude systems to achieve the desired control objective remains an important and challenging research problem.

As a typical nonlinear system, the satellite attitude control system is characterized by complex dynamics, which makes it difficult to establish an accurate mathematical model [7,8,9]. Consequently, model uncertainties are inevitably introduced, including parameter perturbations and unmodeled dynamics [10,11]. In addition, during on-orbit operation, the satellite attitude system is susceptible to external disturbances, such as aerodynamic disturbances [12]. These adverse factors may degrade the control performance of the system and may even compromise its stability [13,14]. To address these issues, a common approach is to employ disturbance observers to estimate external disturbances and mitigate their effects via feed-forward control. For example, refs. [15,16] addressed external disturbance estimation in satellite attitude systems by means of a nonlinear disturbance observer, thereby improving system robustness. Ref. [17] integrates a fuzzy logic system with a nonlinear disturbance observer to address model uncertainties and external disturbances, thereby enhancing the tracking accuracy of the system. In [18], a second-order disturbance observer was developed to address relatively fast-varying disturbances, which consequently enhanced the system control performance. However, the disturbance estimation error of the aforementioned observers requires, in theory, an infinite time to converge. To achieve external disturbance estimation within a finite time, finite-time and fixed-time disturbance observers have attracted considerable research attention. For example, ref. [19] proposed a finite-time disturbance observer to estimate system disturbances, whose estimation error is guaranteed to converge within a finite time. A fixed-time disturbance observer for external disturbance estimation was introduced in [20,21], where the fixed-time convergence of the estimation error is achieved without dependence on the initial conditions. Although the finite-time and fixed-time disturbance observers proposed in [19,20,21] can guarantee disturbance estimation within a finite time, their convergence time cannot be explicitly prescribed in advance. Therefore, achieving accurate disturbance estimation within a user-prescribed time remains a challenging issue and warrants further investigation.

In addition to achieving a stable tracking control of satellite systems, it is often required to satisfy prescribed performance requirements, including both transient and steady-state constraints. To this end, prescribed performance control (PPC), as an effective control strategy for enforcing desired performance constraints, has been widely applied in practical engineering applications. Specifically, an appropriate nonlinear transformation is introduced to reformulate the system subject to constraints into an equivalent constraint-free one. By analyzing the boundedness of the transformed system, sufficient conditions are derived to ensure that the original system satisfies the prescribed performance constraints [22,23,24,25,26]. For example, ref. [27] proposed a robust tracking control strategy for the unmanned helicopter system, where prescribed transient and steady-state performance is guaranteed by imposing a performance funnel constraint on the tracking error dynamics. In [28], an integral barrier Lyapunov function was combined with the PPC method to address the attitude-tracking control problem of rigid satellites, thereby effectively constraining the tracking error. Ref. [29] employed the PPC method to ensure that the satellite attitude-tracking error satisfies prescribed performance constraints, such that the tracking error remains within predefined bounds at all times. Therefore, the PPC-based control strategies have been extensively studied and applied to achieve prescribed tracking performance [30,31,32,33]. However, these elegant methods proposed in [30,31,32,33] do not incorporate disturbance compensation, thereby limiting further improvements in control accuracy and transient performance. Moreover, the aforementioned methods are developed based on the backstepping method, where the introduction of virtual controllers inevitably induces virtual tracking errors. To ensure that tracking errors remain within the prescribed performance envelope, additional performance functions need to be required, inevitably leading to the increasing complexity of the overall controller design.

Inspired by the above analysis, an improved anti-disturbance prescribed performance control method is developed for the rigid satellite attitude system subject to external disturbances. The primary innovations of this paper can be outlined as follows:

- Even though the disturbance observers proposed in the refs. [19,20,21] overcome the theoretical limitation of conventional DOs, whose convergence time of estimation error tends to infinity, the converging time of their estimation errors still cannot be specified in advance. To ensure that the attitude-tracking error can converge within the appointed time, the disturbance observer must accurately estimate the disturbance prior to this time. To address this issue, a prescribed-time disturbance observer (PTDO) is proposed, which guarantees that the disturbance estimation error is convergent within the predefined time.
- To alleviate the design burden introduced by virtual controllers and effectively compensate for the disturbances, this paper proposes the anti-disturbance prescribed performance control scheme for the satellite attitude system. By ensuring the constraints of the constructed sliding-mode error, the desired tracking performance of the satellite attitude system is guaranteed, leading to the fact that the overshoot does not exceed a predefined level and the tracking error is convergent within the appointed time.

The organization of the paper is outlined in what follows. Section 2 presents the problem statement along with necessary preliminaries. In Section 3, a prescribed-time disturbance observer is established. The anti-disturbance prescribed performance controller is addressed in Section 4. Section 5 is dedicated to the stability analysis, and Section 6 concludes this paper.

## 2. Problem Formulation and Preliminaries

According to ref. [28], let $\sigma ={\left[\sigma_{1}\;\sigma_{2}\;\sigma_{3}\right]}^{T}$ and $\omega ={\left[\omega_{1}\;\omega_{2}\;\omega_{3}\right]}^{T}$ denote the attitude angles and the angular velocity, respectively, both defined in the body-fixed coordinate frame with respect to the inertial frame. Then, the rigid satellite attitude dynamics can be described as follows:

(1) $$\begin{array}{c} \left\{\begin{array}{c} \dot{\sigma }=\frac{1}{4}\left[\left(1-\sigma^{T}\sigma \right)I_{3}+2\sigma^{\times }+2\sigma \sigma^{T}\right]\omega =G\left(\sigma \right)\omega \\ J\dot{\omega }=-\omega^{\times }J\omega +Du+d \end{array}\right. \end{array}$$

where $D\in R^{3\times 3}$ is the control allocation matrix. $u\in R^{3\times 1}$ is the control input of the attitude system. *J* is the inertia matrix and $I_{3}\in R^{3\times 3}$ denotes the identity matrix. The terms $\sigma^{\times }$ and $\omega^{\times }\in R^{3\times 3}$ denote the skew–symmetric matrices associated with the vectors σ and ω, respectively, and are given by

$$\sigma^{\times }=\left[\begin{array}{ccc} 0 & -\sigma_{3} & \sigma_{2}\\ \sigma_{3} & 0 & -\sigma_{1}\\ -\sigma_{2} & \sigma_{1} & 0 \end{array}\right],\;\omega^{\times }=\left[\begin{array}{ccc} 0 & -\omega_{3} & \omega_{2}\\ \omega_{3} & 0 & -\omega_{1}\\ -\omega_{2} & \omega_{1} & 0 \end{array}\right].$$

Here, *d* represents the external disturbance acting on the satellite attitude system, denoted as $d={\left[d_{1}\;d_{2}\;d_{3}\right]}^{T}$. Additionally, the rigid satellite attitude system, (1), can be further defined as follows:

(2) $$\begin{array}{c} \left\{\begin{array}{c} \dot{\sigma }=G\left(\sigma \right)\omega \\ \dot{\omega }=-f\left(\omega \right)+g\left(\omega \right)u+\Xi \end{array}\right. \end{array}$$

where $f\left(\omega \right)=J^{-1}\omega^{\times }J\omega$, $g\left(\omega \right)=J^{-1}D$ and $\Xi =J^{-1}d={\left[\Xi_{1}\;\Xi_{2}\;\Xi_{3}\right]}^{T}$.

Prior to the controller development, the rigid satellite attitude system, (1), is required to include the following assumptions and lemmas.

**Assumption** **1** ([18]). *The disturbance signals $\Xi_{i}\left(t\right)$(i=1,2,3) along with their first derivatives are bounded. That is, there exist the constants ${\bar{\xi }}_{i}>0$ and $\xi_{i}>0$ such that $|\Xi_{i}\left(t\right)|\leq {\bar{\xi }}_{i}$ and $|{\dot{\Xi }}_{i}\left(t\right)|\leq \xi_{i}$.*

**Remark** **1.** *It should be noted that the subsequent PTDO design relies on the availability of an upper bound on the disturbance derivative, while no prior knowledge of the disturbance magnitude itself is required. This assumption constitutes one of the key conditions for ensuring that the disturbance can be effectively estimated within the prescribed time. From an engineering perspective, the above assumption is reasonable and practically acceptable. On the one hand, in most physical systems, external disturbances and unmodeled dynamics typically arise from environmental variations and parametric uncertainties. Their evolution processes are generally continuous and constrained by the physical properties of the system as well as its operating conditions. Consequently, the rate of variation of such disturbances is usually bounded under normal operating conditions. On the other hand, although the exact upper bound of the disturbance derivative is difficult to obtain through direct measurement, a conservative estimate can be determined in practice based on the system operating range, known physical limitations, and historical empirical data. Such a conservative bound is sufficient for the observer design and the associated stability analysis, without requiring additional sensors or introducing complex online identification mechanisms. Therefore, introducing the boundedness of the disturbance derivative as a prior assumption is not only physically meaningful but also practically implementable, providing a reasonable and realistic theoretical foundation for the design of the PTDO.*

**Lemma** **1** ([34]). *Consider the following time-varying scaling function:*

(3) $$\begin{array}{c} \nu \left(t\right)=\left\{\begin{array}{c} \frac{P_{t}^{l}}{{\left(P_{t}-t\right)}^{l}},\;\forall \;t\in \left[0,P_{t}\right),\\ 1,\;\;\;\;\;\;\;\forall \;t\in [P_{t},\infty ). \end{array}\right. \end{array}$$

*where $P_{t}>0$ and l>1, with $P_{t}$ being a positive constant that can be specified by the user. Over the interval $\left[0,P_{t}\right)$, the function ν(t) is strictly increasing and satisfies ν(0)=1 and $\lim_{t\to P_{t}^{-}}\nu \left(t\right)=\infty$.*

*The first-order derivative of ν(t) is deduced as*

(4) $$\begin{array}{c} \dot{\nu }\left(t\right)=\left\{\begin{array}{cc} \frac{lP_{t}^{l}}{{\left(P_{t}-t\right)}^{l+1}}, & \forall \;t\in [0,P_{t})\\ 0, & \forall \;t\in [P_{t},\infty ). \end{array}\right. \end{array}$$

*Moreover, by taking the second derivative of ν(t), one obtains*

(5) $$\begin{array}{c} \ddot{\nu }\left(t\right)=\left\{\begin{array}{cc} \frac{l\left(l+1\right)P_{t}^{l}}{{\left(P_{t}-t\right)}^{l+2}}, & \forall \;t\in [0,P_{t})\\ 0, & \forall \;t\in [P_{t},\infty ). \end{array}\right. \end{array}$$

*Then, for $t\in [0,P_{t})$, $\frac{\dot{\nu }}{\nu }=\frac{l}{P_{t}-t}$ and $\frac{\ddot{\nu }}{\dot{\nu }}=\frac{l(l+1)}{{\left(P_{t}-t\right)}^{2}}$ can be obtained.*

**Lemma** **2** ([34]). *Consider a nonlinear time-varying system described by $\dot{x}\left(t\right)=\phi \left(t,x(t)\right)$. Suppose that there exists a positive definite Lyapunov function V(x) such that for all t≥0, the following inequality holds:*

(6) $$\dot{V}\left(t\right)\leq -\alpha V\left(t\right)-2\frac{\dot{\nu }\left(t\right)}{\nu (t)}V\left(t\right),$$

*where α is a positive constant. Under this condition, it can be shown that for $t\in [0,P_{t})$, $V\left(t\right)\leq \nu^{-2}\left(t\right)e^{-\alpha t}V\left(0\right)$, whereas for $t\in [P_{t},\infty )$ the Lyapunov function becomes identically zero, i.e., V(t)≡0. This implies that the system states converge to the origin no later than the prescribed time $P_{t}$ defined in (3), thereby guaranteeing global prescribed-time stability. Moreover, if the term (6) can be deduced as*

(7) $$\begin{array}{c} \dot{V}\leq -\alpha V-2\frac{\dot{\nu }}{\nu }V+\tau , \end{array}$$

*where τ>0, then practical prescribed-time stability is ensured. In this case, the system trajectories converge to a residual set whose ultimate bound is given by $\frac{\tau }{\alpha }$.*

**Lemma** **3** ([18]). *Consider the dynamical system $\dot{x}=f\left(x\right)$. Suppose that there exists a continuous Lyapunov function V(x) that is positive definite and satisfies the inequalities $\alpha_{1}{(∥x∥}_{F})\leq V\left(x\right)\leq \alpha_{2}{(∥x∥}_{F})$, where $\alpha_{1}\left(\cdot \right)$ and $\alpha_{2}\left(\cdot \right)$ are class-K functions. Furthermore, assume that the derivative of V(x) obeys $\dot{V}\left(x\right)\leq -\beta_{1}V\left(x\right)+\beta_{2}$, with constants $\beta_{1}>0$ and $\beta_{2}>0$. Under these conditions, the state x(t) remains uniformly ultimately bounded.*

## 3. Disturbance Estimation Based on the PTDO

In System (2), we consider the external disturbance $\Xi \in R^{3}$ defined as $\Xi =J^{-1}d={\left[\Xi_{1}\;\Xi_{2}\;\Xi_{3}\right]}^{T}$. The objective is to estimate each disturbance element $d_{i}$(i=1,2,3) within its corresponding predefined time $P_{ti}$. To this end, the PTDO is developed as

(8) $$\begin{array}{c} \left\{\begin{array}{c} {\dot{\hat{\omega }}}_{i}=-f_{i}\left(\omega \right)+g_{i}\left(\omega \right)u_{i}+{\hat{\Xi }}_{i}\\ {\hat{\Xi }}_{i}=-\left(P_{1,i}+P_{2,i}\frac{\dot{\nu_{i}}}{\nu_{i}}\right){\tilde{\omega }}_{i}-\int_{0}^{t}\xi_{i}\mathrm{sign}\left({\tilde{\omega }}_{i}\right)ds, \end{array}\right. \end{array}$$

where $u_{i}$, ${\hat{\omega }}_{i}$, and ${\hat{\Xi }}_{i}$ represent the estimated values corresponding to the *i*-th elements of *u*, ω, and Ξ, respectively. The parameters $P_{1,i}$, $P_{2,i}$, and $\xi_{i}$ are positive constants satisfying $P_{1,i}>\frac{1}{2}$ and $P_{2,i}>1$. Define the estimation errors as ${\tilde{\omega }}_{i}={\hat{\omega }}_{i}-\omega_{i}$ and ${\tilde{\Xi }}_{i}={\hat{\Xi }}_{i}-\Xi_{i}$. Then, the derivative of ${\tilde{\omega }}_{i}$ is given by

(9) $$\begin{array}{c} {\dot{\tilde{\omega }}}_{i}={\dot{\hat{\omega }}}_{i}-{\dot{\omega }}_{i}=\left(f_{i}\left(\omega \right)+g_{i}\left(\omega \right)u_{i}+{\hat{\Xi }}_{i}\right)-\left(f_{i}\left(\omega \right)+g_{i}\left(\omega_{i}\right)u_{i}+\Xi_{i}\right)={\hat{\Xi }}_{i}-\Xi_{i}. \end{array}$$

Based on (8), taking the derivative of ${\tilde{\omega }}_{i}$ yields

(10) $$\begin{array}{c} \left\{\begin{array}{c} {\dot{\tilde{\omega }}}_{i}=-\left(P_{1,i}+P_{2,i}\frac{{\dot{\nu }}_{i}}{\nu_{i}}\right){\tilde{\omega }}_{i}-\varphi_{i}\left(t\right)\\ \varphi_{i}\left(t\right)=\int_{0}^{t}\xi_{i}\mathrm{sign}\left({\tilde{\omega }}_{i}\right)ds+\Xi_{i}, \end{array}\right. \end{array}$$

where $\varphi_{i}\left(t\right)$ is an auxiliary intermediate variable. Moreover, Proposition 1 provides a sufficient condition under which the PTDO (8) guarantees prescribed-time disturbance estimation.

**Proposition** **1.** *The observer (8) achieves prescribed-time estimation of each disturbance component $\Xi_{i}\;\left(i=1,2,3\right)$ in System (4) when the design parameters satisfy*

(11) $$\begin{array}{c} P_{1,i}>\frac{1}{2},\;P_{2,i}>1,\;\xi_{i}>\left|{\dot{\Xi }}_{i}\right|. \end{array}$$

*Thus, the estimation error ${\tilde{\Xi }}_{i}$ reaches an invariant neighborhood of the origin and remains therein no later than the prescribed time $P_{ti}$.*

**Proof.**  For i=1,2,3, we construct the Lyapunov function as

(12) $$\begin{array}{c} V_{i}=\frac{1}{2}{\tilde{\omega }}_{i}^{2}. \end{array}$$

Taking the derivative of $V_{i}$ along System (10) yields

(13) $$\begin{array}{c} {\dot{V}}_{i}=-\left(P_{1,i}+P_{2,i}\frac{{\dot{\nu }}_{i}}{\nu_{i}}\right){\tilde{\omega }}_{i}^{2}-{\tilde{\omega }}_{i}\varphi_{i}. \end{array}$$

When ${\tilde{\omega }}_{i}>0$, it follows that $\mathrm{sign}(\omega_{i})=1$. In conjunction with (11) and Assumption 1, one can obtain ${\dot{\varphi }}_{i}\left(t\right)=\xi_{i}+{\dot{\Xi }}_{i}\geq 0$. From (10), it is observed that $\varphi_{i}\left(0\right)=\Xi_{i}\left(0\right)$, where $\Xi_{i}\left(0\right)$ corresponds to the value of the disturbance $\Xi_{i}\left(t\right)$ at the initial time. Consequently, one has ${\tilde{\omega }}_{i}\varphi_{i}\left(t\right)\geq {\tilde{\omega }}_{i}\Xi_{i}\left(0\right)$. For the case ${\tilde{\omega }}_{i}<0$, an analogous argument yields the same result. Therefore, for all t≥0, one has ${\tilde{\omega }}_{i}\varphi_{i}\left(t\right)\geq {\tilde{\omega }}_{i}\Xi_{i}\left(0\right)$. Thus, the derivative of $V_{i}$ is given by

(14) $$\begin{array}{ccc} {\dot{V}}_{i} & \leq & -2P_{1,i}V_{i}-2P_{2,i}\frac{{\dot{\nu }}_{i}}{\nu_{i}}V_{i}-{\tilde{\omega }}_{i}\Xi_{i}\left(0\right)\\ & \leq & -\left(2P_{1,i}-1\right)V_{i}-2P_{2,i}\frac{{\dot{\nu }}_{i}}{\nu_{i}}V_{i}+\frac{1}{2}{\bar{\xi }}_{i}^{2}. \end{array}$$

Furthermore, an equivalent representation of (14) is deduced as

(15) $$\begin{array}{c} {\dot{V}}_{i}\leq -\left(2P_{1,i}-1\right)V_{i}-2P_{2,i}\frac{\dot{\nu_{i}}}{\nu_{i}}V_{i}+\frac{1}{2}{\bar{\xi }}_{i}^{2}. \end{array}$$

Then, according to Lemma 2 and (15), it follows that $|{\tilde{\omega }}_{i}\left(t\right)|\leq \frac{{\bar{\xi }}_{i}}{\sqrt{2P_{1,i}-1}}$ for all $t\geq P_{ti}$. Consequently, the estimation error ${\tilde{\Xi }}_{i}={\hat{\Xi }}_{i}-\Xi_{i}={\dot{\tilde{\omega }}}_{i}\;\left(i=1,2,3\right)$, approaches a bounded residual set within the prescribed time $P_{ti}$. This completes the proof. □

**Remark** **2.** *Based on prescribed-time stability theory, this paper proposes the PTDO to estimate external disturbances in rigid satellite attitude systems. The proposed observer ensures that the disturbance estimation error is confined to a small neighborhood of the origin within an arbitrarily prescribed time. Compared with the observer designs reported in the ref. [19,20,21], the proposed observer contains a simpler structure, and the convergence time can be specified in advance.*

## 4. Disturbance-Observed Prescribed Performance Controller

In this section, an anti-disturbance control scheme is proposed by integrating the PPC with the proposed PTDO, such that the tracking objective is fulfilled and the prescribed requirements on both transient and steady-state performance are satisfied. First, an appointed-time performance function (ATPF) is given by

(16) $$\begin{array}{c} \eta_{i}\left(t\right)=\left\{\begin{array}{c} {\left(1-\frac{t}{A_{ti}}\right)}^{\iota_{i}}\left(\eta_{0i}-\eta_{\infty i}\right)+\eta_{\infty i},\;t<A_{ti},\\ \eta_{\infty i},\;\;\;\;\;\;\;\;\;\;\;t\geq A_{ti}, \end{array}\right. \end{array}$$

where $\eta_{0i}$ and $\eta_{\infty i}$ denote the initial and steady-state values of the ATPF, respectively, and $\iota_{i}>2$ is a design parameter employed to regulate the convergence characteristics and smoothness of the function $\eta_{i}\left(t\right)$. Moreover, $A_{ti}$ represents a user-defined time parameter, which can be arbitrarily chosen. By computing the derivative of $\eta_{i}\left(t\right)$, one obtains

(17) $$\begin{array}{c} {\dot{\eta }}_{i}\left(t\right)=\left\{\begin{array}{c} -\frac{\iota_{i}}{A_{ti}}{\left(1-\frac{t}{A_{ti}}\right)}^{\iota_{i}-1}\left(\eta_{0i}-\eta_{\infty i}\right),\;t<A_{ti}\\ 0,\;\;\;\;\;\;\;\;\;\;\;t\geq A_{ti}. \end{array}\right. \end{array}$$

Let $e_{\sigma i}$ and $e_{\upsilon i}$ denote the tracking error and the sliding-mode error, respectively, given by

(18) $$\begin{array}{c} \left\{\begin{array}{c} e_{\sigma i}=\sigma_{i}-\sigma_{ri},\\ e_{\upsilon i}={\dot{e}}_{\sigma i}+\lambda_{i}e_{\sigma i}, \end{array}\right. \end{array}$$

where $\sigma_{i}$ denotes the *i*-th component of the actual attitude angle, and $\sigma_{ri}$ denotes the *i*-th component concerning the reference signal. The admissible evolution of the constructed error $e_{\upsilon i}$ is characterized by the prescribed performance relations, expressed as

(19) $$\begin{array}{c} {\underline{\eta }}_{i}\left(t\right)<e_{\upsilon i}\left(t\right)<{\bar{\eta }}_{i}\left(t\right), \end{array}$$

where the function ${\underline{\eta }}_{i}\left(t\right)$ is a continuous and strictly negative monotonically increasing function, whereas the function ${\bar{\eta }}_{i}\left(t\right)$ is a continuous and strictly positive monotonically decreasing function. Their specific forms are deduced as

(20) $${\underline{\eta }}_{i}\left(t\right)=\left\{\begin{array}{cc} {\left(1-\frac{t}{A_{ti}}\right)}^{\iota_{i}}\left({\underline{\eta }}_{0i}-{\underline{\eta }}_{\infty i}\right)+{\underline{\eta }}_{\infty i}, & t<A_{ti},\\ {\underline{\eta }}_{\infty i}, & t\geq A_{ti}, \end{array}\right.$$

and

(21) $${\bar{\eta }}_{i}\left(t\right)=\left\{\begin{array}{cc} {\left(1-\frac{t}{A_{ti}}\right)}^{\iota_{i}}\left({\bar{\eta }}_{0i}\left(t_{k}\right)-{\bar{\eta }}_{\infty i}\right)+{\bar{\eta }}_{\infty i}, & t<A_{ti},\\ {\bar{\eta }}_{\infty i}, & t\geq A_{ti}. \end{array}\right.$$

To ensure that the overshoot of the sliding-mode error remains within the specified threshold $\mu_{i}>0$, then ${\underline{\eta }}_{0i}$ and ${\bar{\eta }}_{0i}$ must satisfy the following conditions:

(22) $$\begin{array}{ccc} & & e_{\upsilon i}\left(0\right)>0\Rightarrow {\bar{\eta }}_{0i}>e_{\upsilon i}\left(0\right)\wedge {\underline{\eta }}_{0i}=-\mu_{i}, \end{array}$$

(23) $$\begin{array}{ccc} & & e_{\upsilon i}\left(0\right)<0\Rightarrow {\underline{\eta }}_{0i}<e_{\upsilon i}\left(0\right)\wedge {\bar{\eta }}_{0i}=\mu_{i}, \end{array}$$

(24) $$\begin{array}{ccc} & & e_{\upsilon i}\left(0\right)=0\Rightarrow {\bar{\eta }}_{0i}=\mu_{i}\wedge {\underline{\eta }}_{0i}=-\mu_{i}. \end{array}$$

Moreover, it follows from (19) that the tracking error $e_{\sigma }=\sigma -\sigma_{r}$ satisfies implicit performance constraints. Consequently, for the constructed error $e_{\upsilon i}\left(t\right)$ in (18), the output is given by

(25) $$\begin{array}{c} e_{\sigma i}\left(t\right)=e_{\sigma i}\left(0\right)\exp\left(-\lambda_{i}t\right)+\int_{0}^{t}e_{\upsilon i}\left(s,e_{\sigma i}\left(s\right),{\dot{e}}_{\sigma i}\left(s\right)\right)\exp\left(-\lambda_{i}\left(t-s\right)\right)\;ds. \end{array}$$

According to the term (25), one can obtain that

(26) $$\begin{array}{ccc} \left|e_{\sigma i}\left(t\right)\right| & \leq & \left|e_{\sigma i}\left(0\right)\right|\exp\left(-\lambda_{i}t\right)+\left|e_{\upsilon i}\left(t\right)\right|\int_{0}^{t}\exp\left(-\lambda_{i}\left(t-s\right)\right)ds\\ & \leq & \left|e_{\sigma i}\left(0\right)\right|\exp\left(-\lambda_{i}t\right)+\frac{1}{\lambda_{i}}\left[1-\exp(-\lambda_{i}t)\right]\max\left\{|{\underline{\eta }}_{i}|,{\bar{\eta }}_{i}\right\}. \end{array}$$

Then, as a consequence, the evolution of the error signal $e_{\sigma i}\left(t\right)$ is governed by the prescribed performance (26) in an implicit manner. Moreover, with a proper choice of the design parameters $\lambda_{i}>1$(i=1,2,3), the tracking error can be approximately driven into a bounded set within the appointed time $A_{ti}$.

**Remark** **3.** *From (26), it can be observed that eliminating the exponential term $\exp(-\lambda_{i}t)$ allows error signal $e_{\sigma i}\left(t\right)$ to reach a bounded region within the appointed time $A_{ti}$, while yielding a more stringent performance bound. Nevertheless, the inclusion of the term $\exp(-\lambda_{i}t)$ degrades the convergence behavior of the tracking error. Noting that increasing the parameter $\lambda_{i}$ enhances the decay rate of the exponential term, such that $\exp(-\lambda_{i}t)$ diminishes to a negligible magnitude over a short duration. Consequently, error signal $e_{\sigma i}\left(t\right)$ can be regarded as being approximately confined to a bounded region within the appointed time $A_{ti}$.*

Prior to the transformation of the error constraint into an unconstrained one, the asymmetric performance constraint (19) is first transformed into a symmetric form, and the corresponding transformation procedure is given in Proposition 2.

**Proposition** **2.** *For $\eta_{i}\left(t\right)$ and $\eta_{i}\left(t\right)$ given in (20) and (21), respectively, the asymmetric performance constraint (19) is equivalently deduced as*

(27) $$\begin{array}{c} -\eta_{i}^{r}\left(t\right)<e_{\bar{\upsilon }i}\left(t\right)<\eta_{i}^{r}\left(t\right), \end{array}$$

*where* $e_{\bar{\upsilon }i}\left(t\right)=e_{\upsilon i}\left(t\right)-\frac{1}{2}\left[{\underline{\eta }}_{i}\left(t\right)+\bar{\eta }\left(t\right)\right]$ *and* $\eta_{i}^{r}\left(t\right)=\frac{1}{2}\left[{\bar{\eta }}_{i}\left(t\right)-{\underline{\eta }}_{i}\left(t\right)\right]$.

**Proof.**  By subtracting $\frac{1}{2}\left[{\underline{\eta }}_{i}\left(t\right)+{\bar{\eta }}_{i}\left(t\right)\right]$ from the performance constraint (19), the symmetric performance constraint (27) is obtained, thereby completing the proof. □

Now, based on Proposition 2, the error transformation function is given by

(28) $$\begin{array}{c} F\left(\chi \right)=\frac{\exp(\chi )-1}{\exp(\chi )+1}, \end{array}$$

where F(χ) is a strictly increasing function that satisfies the inequality −1<F(χ)<1, together with the limits $\lim_{\chi \to -\infty }F\left(\chi \right)=-1$ and $\lim_{\chi \to +\infty }F\left(\chi \right)=1$. Consequently, the performance constraint (27) is equivalently deduced as

(29) $$\begin{array}{c} -1<\frac{e_{\bar{\upsilon }i}}{\eta_{i}^{r}}<1. \end{array}$$

Thus, the following expression can be established as

(30) $$\begin{array}{c} e_{\bar{\upsilon }i}\left(t\right)=\eta_{i}^{r}\left(t\right)F\left(\chi_{i}\right). \end{array}$$

Moreover, the inverse function of $F(\chi_{i})$ can be expressed as

(31) $$\begin{array}{c} \chi_{i}=F^{-1}\left(\frac{e_{\bar{\upsilon }i}\left(t\right)}{\eta_{i}^{r}\left(t\right)}\right)=\ln\left(\frac{e_{\bar{\upsilon }i}/\eta_{i}^{r}+1}{1-e_{\bar{\upsilon }i}/\eta_{i}^{r}}\right). \end{array}$$

By computing the derivative of $\chi_{i}$, one obtains

(32) $$\begin{array}{c} {\dot{\chi }}_{i}=r_{i}\left[{\dot{e}}_{\bar{\upsilon }i}-F\left(\chi_{i}\right){\dot{\eta }}_{i}^{r}\right]=r_{i}\left[{\ddot{\sigma }}_{i}-{\ddot{\sigma }}_{ri}+\lambda_{i}{\dot{e}}_{\sigma i}-\frac{1}{2}\left({\dot{\underline{\eta }}}_{i}+{\dot{\bar{\eta }}}_{i}\right)-F\left(\chi_{i}\right){\dot{\eta }}_{i}^{r}\right], \end{array}$$

where $r_{i}=\frac{2}{\eta_{i}^{r}\left(1+e_{\bar{\upsilon }i}/\eta_{i}^{r}\right)\left(1-e_{\bar{\upsilon }i}/\eta_{i}^{r}\right)}$. The term (32) can be further rewritten as

(33) $$\begin{array}{ccc} \dot{\chi } & = & R\left[\ddot{\sigma }-{\ddot{\sigma }}_{r}+\bar{\lambda }{\dot{e}}_{\sigma }-\bar{\eta }-\bar{F}\left(\chi \right){\dot{\eta }}^{r}\right]\\ & = & R\left\{G\left(\dot{\sigma }\right)\omega +G\left(\sigma \right)\left[-f(\omega )+g(\omega )u+\Xi \right]-{\ddot{\sigma }}_{r}+\bar{\lambda }{\dot{e}}_{\sigma }-\bar{\eta }-\bar{F}\left(\chi \right){\dot{\eta }}^{r}\right\}\\ & = & R\left\{G\left(\dot{\sigma }\right)\omega +G\left(\sigma \right)\left[-f(\omega )+g(\omega )u+\Xi \right]+\Pi \left(t\right)-\bar{\eta }-\bar{F}\left(\chi \right){\dot{\eta }}^{r}\right\}, \end{array}$$

where $\chi ={\left[\chi_{1},\chi_{2},\chi_{3}\right]}^{T}$, $\Pi \left(t\right)=-{\ddot{\sigma }}_{r}+\bar{\lambda }{\dot{e}}_{\sigma }$, $\bar{F}\left(\chi \right)=\mathrm{diag}\left\{F\left(\chi_{1}\right),F\left(\chi_{2}\right),F\left(\chi_{3}\right)\right\}$, ${\dot{\eta }}^{r}={\left[{\dot{\eta }}_{1}^{r},{\dot{\eta }}_{2}^{r},{\dot{\eta }}_{3}^{r}\right]}^{T}$, $\bar{\lambda }=\mathrm{diag}\left\{\lambda_{1},\lambda_{2},\lambda_{3}\right\}>0$, $\bar{\eta }=-\frac{1}{2}{\left[{\dot{\underline{\eta }}}_{1}+{\dot{\bar{\eta }}}_{1},{\dot{\underline{\eta }}}_{2}+{\dot{\bar{\eta }}}_{2},{\dot{\underline{\eta }}}_{3}+{\dot{\bar{\eta }}}_{3}\right]}^{T}$, and $R\left(t\right)=\mathrm{diag}\left\{r_{1}\left(t\right),r_{2}\left(t\right),r_{3}\left(t\right)\right\}>0$.

Therefore, based on the disturbance estimation given by (8) and the unconstrained error defined in (33), the following controller is proposed as follows:

(34) $$\begin{array}{c} u=g{\left(\omega \right)}^{-1}\left\{f\left(\omega \right)-\hat{\Xi }+G^{-1}\left(\sigma \right)\left[-G\left(\dot{\sigma }\right)\omega -\Pi \left(t\right)+\bar{\eta }+\bar{F}\left(\chi \right){\dot{\eta }}^{r}-R^{-1}K_{c}\chi \right]\right\}. \end{array}$$

where $K_{c}\in R^{3\times 3}$ is a positive definite matrix to be decided. By combining (33) and (34), one obtains

(35) $$\begin{array}{c} \dot{\chi }=-K_{c}\chi -\tilde{\Xi }. \end{array}$$

**Remark** **4.** *The convergence time $P_{ti}$ of the PTDO in (8) and the convergence time $A_{ti}$ of the ATPF in (16) can both be arbitrarily preassigned at the design stage. However, from a theoretical perspective, the PTDO and the ATPF play different roles in the closed-loop system, which imposes a necessary temporal relationship between their convergence times. Specifically, the PTDO is employed to estimate the external disturbance in the attitude system, and its estimation error is required to converge within a relatively short time so as to provide reliable disturbance compensation for the subsequent control law. In contrast, the ATPF is introduced to regulate the convergence behavior of the tracking error, where the convergence time $A_{ti}$ determines the preassigned time within which the error is driven into a bounded set. If the convergence speed of the PTDO is slower than that of the ATPF, namely $P_{ti}>A_{ti}$, the disturbance estimation error may remain significant during the transient phase of error convergence. As a consequence, the effectiveness of disturbance compensation would be degraded, which may adversely affect the system stability and transient performance. Therefore, to ensure that the control action is consistently based on an accurate disturbance estimation throughout the convergence process, the convergence time of the PTDO should not exceed that of the ATPF, leading to the condition $P_{ti}\leq A_{ti},\;\left(i=1,2,3\right)$. Under this condition, the proposed anti-disturbance controller (35) is able to guide the system states based on sufficiently established disturbance estimation, thereby guaranteeing system stability and ensuring that the tracking error satisfies the desired performance requirements within the appointed time.*

Normally, proper selection of the controller-related parameters is essential to maintain bounded and well-behaved attitude-tracking responses. Therefore, several parameter restrictions are introduced to support the subsequent stability analysis, which are summarized as follows:

(1) To reduce the impact of the term $e_{\sigma i}\left(t\right)$ on the evolution of the tracking error $e_{\sigma i}\left(t\right)$, the parameter $\lambda_{i}$ should satisfy

(36) $$\begin{array}{c} \lambda_{i}>1,\;\forall \;i=1,2,3. \end{array}$$

(2) The parameters ${\underline{\eta }}_{0i}$ and ${\bar{\eta }}_{0i}\;\left(i=1,2,3\right)$ should satisfy

(37) $$\begin{array}{ccc} & & e_{\upsilon i}\left(0\right)>0\Rightarrow {\bar{\eta }}_{0i}>e_{\upsilon i}\wedge {\underline{\eta }}_{0i}=-\mu_{i},\\ & & e_{\upsilon i}\left(0\right)<0\Rightarrow {\underline{\eta }}_{0i}<e_{\upsilon i}\wedge {\bar{\eta }}_{0i}=\mu_{i},\\ & & e_{\upsilon i}\left(0\right)=0\Rightarrow {\bar{\eta }}_{0i}=\mu_{i}\wedge {\underline{\eta }}_{0i}=-\mu_{i}. \end{array}$$

(3) Choose $l_{1}>0$, $l_{2}>0$, and $l_{3}>0$ such that

(38) $$\begin{array}{c} l_{1}{\left(\chi_{1}^{0}\right)}^{2}+l_{2}{\left(\chi_{2}^{0}\right)}^{2}+l_{3}{\left(\chi_{3}^{0}\right)}^{2}<1. \end{array}$$

where $\chi_{i}^{0}$ denotes the initial value of the unconstrained error $\chi_{i}\;\left(i=1,2,3\right)$.

As discussed in the previous subsection, the condition (11) ensures that ${\tilde{\Xi }}_{i}$ and ${\tilde{\omega }}_{i}$ converge to a sufficiently small neighborhood of the origin within the prescribed time $T_{pi}$. Furthermore, based on Proposition 2, the boundedness of $\chi_{i}$ guarantees the validity of (27), which in turn ensures that the performance constraints in (19) are satisfied. The proof of the boundedness of $\chi_{i}$ is given in what follows.

By establishing the boundedness of the unconstrained error χ, condition (27) can be ensured, from which it follows that the error $e_{\bar{\upsilon }i}\left(t\right)$ remains within performance constraints specified in (19). For the purpose of analyzing the stability of the tracking error system, one introduces the Lyapunov function, defined as

(39) $$\begin{array}{c} V_{\chi }=\frac{1}{2}\chi^{T}\chi . \end{array}$$

whose derivative is given by

(40) $$\begin{array}{ccc} {\dot{V}}_{\chi } & = & -\chi^{T}K_{c}\chi -\chi^{T}\tilde{\Xi }\\ & \leq & -\lambda_{\min}\left(K_{c}-\frac{1}{2}I\right)\chi^{T}\chi +\frac{1}{2}{\tilde{\Xi }}^{T}\tilde{\Xi } \end{array}$$

To guarantee the boundedness of the unconstrained error χ, the controller parameter needs to satisfy the following condition:

(41) $$\begin{array}{c} K_{c}>\frac{1}{2}I. \end{array}$$

**Remark** **5.** *The PTDO is developed to estimate the external disturbance, thereby enabling high-precision estimation of the disturbance within an arbitrarily prescribed time. Subsequently, the ATPF is constructed and incorporated into the PPC method, such that the error signals satisfy desired performance, thereby guaranteeing the convergence of the tracking error within the appointed time. Unlike prescribed-time stability theory, which typically requires the constructed Lyapunov function satisfying strict differential inequality conditions to ensure prescribed-time convergence, the PPC approach achieves the aforementioned objective without imposing such requirements, while simultaneously ensuring favorable transient performance of the system. In addition, by constructing the sliding-mode error and enforcing corresponding performance constraints, the tracking error is constrained to remain within the prescribed bounds. This framework circumvents the design of virtual control signals and additional performance function, thereby further simplifying the overall control architecture.*

## 5. Closed-Loop Stability Analysis

According to the analytical results obtained above, this section summarizes the control parameter design method in Theorem 1.

**Theorem** **1.** *For System (1), under the PTDO designed in (8) and the controller given in (35), if there exists positive parameters $P_{1,i},\;\xi_{i},\;P_{ti}$, $\iota_{i}=3,\;A_{ti},\;{\underline{\beta }}_{i},\;{\bar{\beta }}_{i},\;\mu_{i},\;\lambda_{i}\;\left(i=1,2,3\right)$, and $K_{c}$ that satisfy the conditions (36), (37), (38) and (41), then the estimation error ${\tilde{\Xi }}_{i}$ is driven into a bounded set within the prescribed time $P_{ti}$. Moreover, the tracking error $e_{\upsilon i}\left(t\right)$ complies with the prescribed performance constraint (26) and approaches the steady-state interval $\left({\underline{\eta }}_{\infty i},\;{\bar{\eta }}_{\infty i}\right)$ within the appointed time $A_{ti}$.*

**Proof.**  Based on (12) and (39), the following Lyapunov function is adopted:

(42) $$\begin{array}{c} V=\sum_{i=1}^{3}V_{i}+V_{\chi }. \end{array}$$

Differentiating (42) with respect to time yields

(43) $$\begin{array}{ccc} \dot{V} & = & \sum_{i=1}^{3}{\dot{V}}_{i}+{\dot{V}}_{\chi }\\ & \leq & -\left(2P_{1,i}-1\right)V_{i}-2P_{2,i}\frac{\dot{\nu_{i}}}{\nu_{i}}V_{i}+\frac{1}{2}{\bar{\xi }}_{i}^{2}-\lambda_{\min}\left(2K_{c}-I\right)V_{\chi }+\frac{1}{2}{\tilde{\Xi }}^{T}\tilde{\Xi }\\ & \leq & -\min\left\{B_{i},\lambda_{\min}\left(2K_{c}-I\right)\right\}V+\gamma , \end{array}$$

where $B_{i}=2P_{1,i}-1+2P_{2,i}\frac{\dot{\nu_{i}}}{\nu_{i}}$ and $\gamma =\frac{1}{2}{\bar{\xi }}_{i}^{2}+\frac{1}{2}{\tilde{\Xi }}^{T}\tilde{\Xi }$. Therefore, according to Lemma 3, the error variables $\tilde{\Xi }$ and χ remain bounded over time, which concludes the proof. □

**Remark** **6.** *Building upon the proposed control framework and theoretical investigation, this paper presents several innovations. First, the PTDO-based approach is developed to address disturbances in the attitude system within the prescribed time. Second, by incorporating the PPC control strategy, the error signals are ensured to evolve within the prescribed performance bounds, such that the overshoot does not exceed the predefined level and attitude-tracking error approximately converges to a sufficiently small neighborhood around zero within the appointed time.*

## 6. Simulated Example

Simulation results are shown in this section to evaluate the feasibility of the proposed control method. Initially, as for System (1), the inertia matrix is chosen as

$$J=\left[\begin{array}{ccc} 20 & 1.2 & 0.9\\ 1.2 & 17 & 1.4\\ 0.9 & 1.4 & 15 \end{array}\right]\;\left(\mathrm{kg}\cdot m^{2}\right),$$

with the desired attitude angle set as $\sigma_{r}={\left[0\;0\;0\right]}^{T}$(rad). The initial conditions for the attitude angle and angular velocity are taken as $\sigma \left(0\right)={\left[0.2\;0.1\;0.2\right]}^{T}$(rad) and $\omega \left(0\right)={\left[0\;0\;0\right]}^{T}\left(\mathrm{rad}/s\right)$, respectively. The control allocation matrix is defined as $D=I_{3}$, and the external disturbance is considered as

$$\begin{array}{c} \Xi \left(t\right)=\left[\begin{array}{c} 0.6\cos(2t+0.4)+0.4\sin(1.5t+0.4)\\ 1.2\cos(2t+0.4)-0.4\cos(1.5t+0.4)\\ \sin(2t+0.4)+0.2\cos(1.5+0.4) \end{array}\right]\left(\mathrm{rad}/s^{2}\right). \end{array}$$

**Figure 1:**
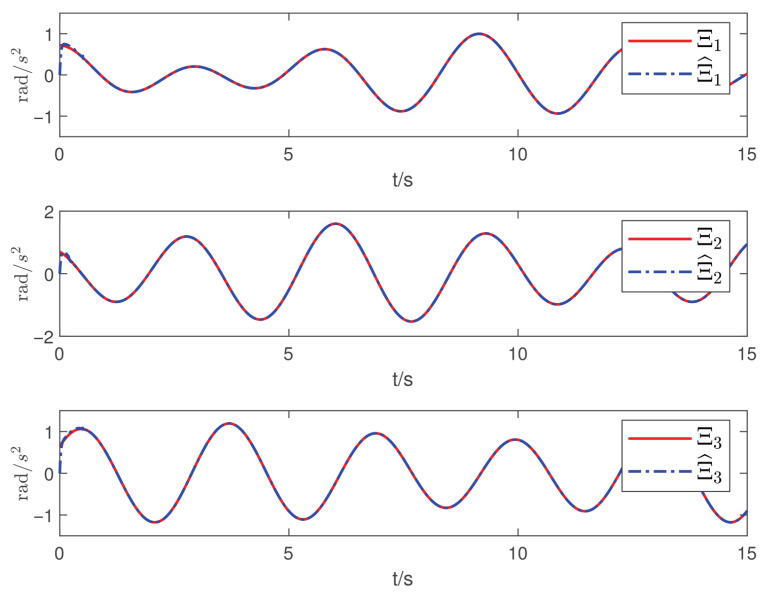
Tracking responses of the disturbance estimation.

**Figure 2:**
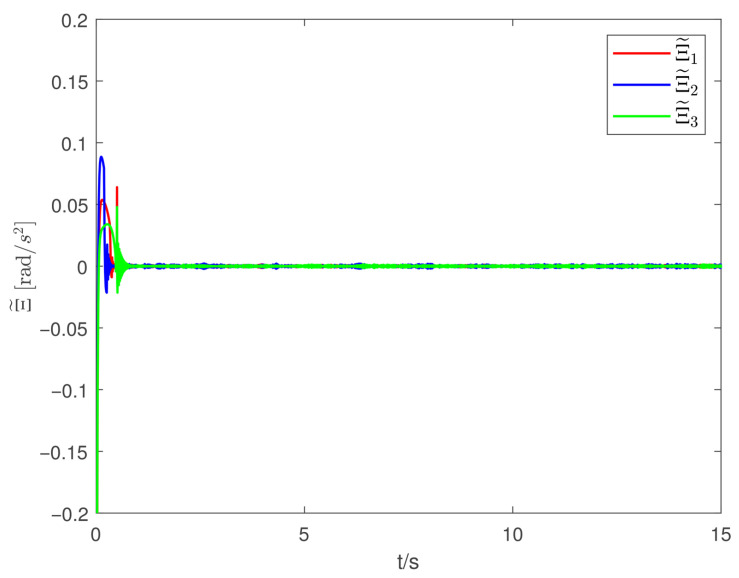
Tracking responses of the disturbance estimation error.

**Figure 3:**
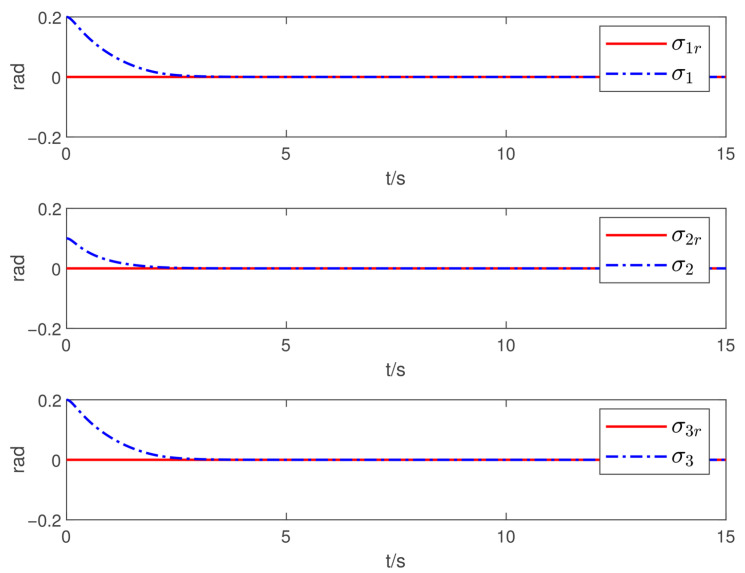
Tracking responses of the attitude angle.

**Figure 4:**
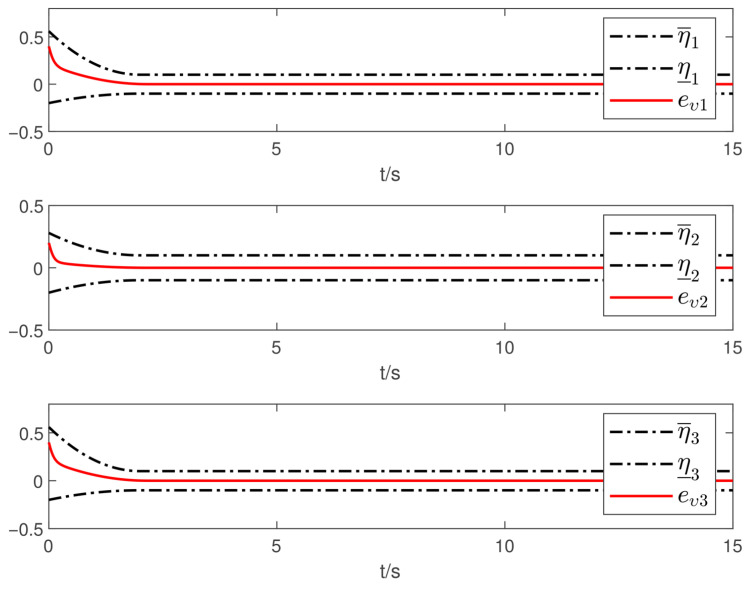
Responses of the sliding-mode error.

**Figure 5:**
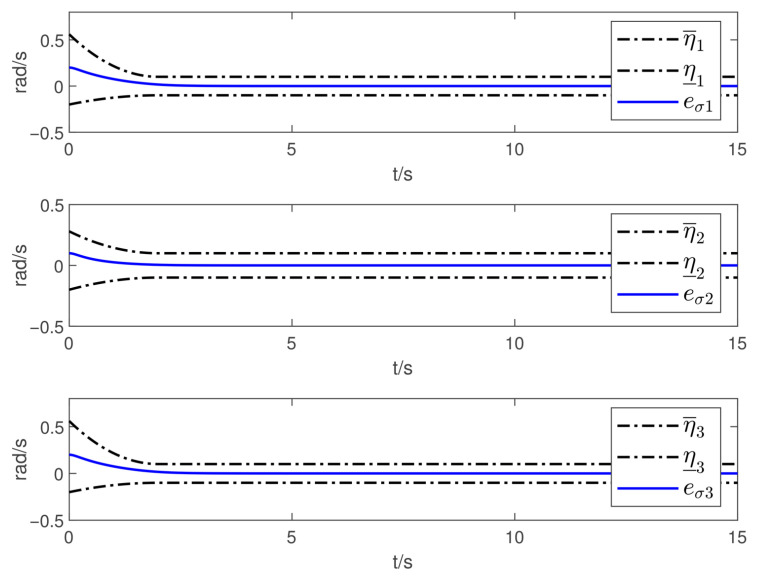
Responses of the attitude-tracking error.

**Figure 6:**
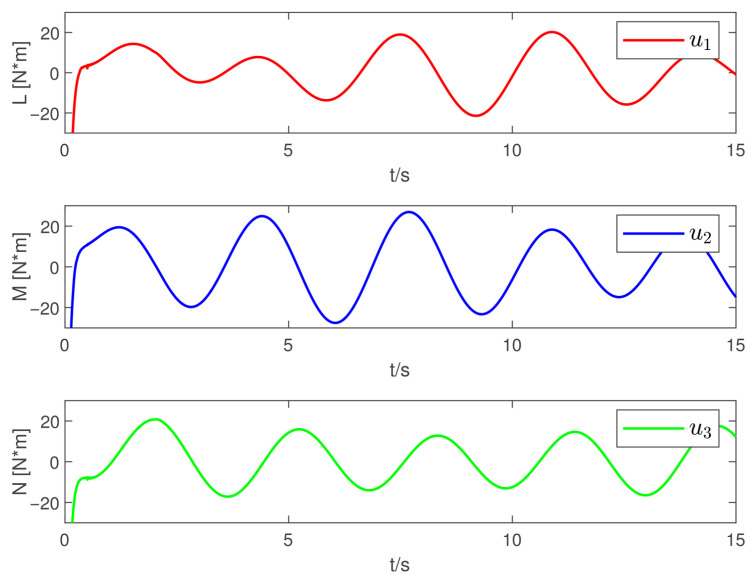
Responses of the control force moments.

Now, according to Proposition 1 and Theorem 1, the observer and controller parameters must satisfy the conditions given in (11), (36), (37), (38), and (41), which ensures that all closed-loop error signals are uniformly ultimately bounded and that the desired tracking performance is achieved. To this end, to demonstrate the effectiveness of the proposed PTDO, its parameters are selected as $P_{1,i}\;\left(i=1,2,3\right)=20,\;P_{2,i}=3,\;\xi_{1}=1.8,\;\xi_{2}=3.0,\;\xi_{3}=2.3,\;l_{i}=3,$ and $P_{ti}=0.5.$ For the PPC-based controller, the parameters are chosen as $\iota_{i}=3,\;A_{ti}=2,\;{\underline{\beta }}_{i}={\bar{\beta }}_{i}=1.2,\;\mu_{i}=0.1,\;\lambda_{i}=2\;\left(i=1,2,3\right),$ and $K_{c}=\mathrm{diag}\left\{10,\;10,\;10\right\}.$ It is evident that the above parameter selections satisfy the conditions in (11), (36), (37), (38), and (41). The relevant simulation results are presented in Figure 1, Figure 2, Figure 3, Figure 4, Figure 5 and Figure 6.

Figure 1 shows the estimation results of the external disturbances, demonstrating that the PTDO proposed in (8) achieves accurate disturbance estimation within the prescribed time. Correspondingly, Figure 2 illustrates the response of the disturbance estimation error, which can rapidly converge to a sufficiently small bound within 0.5 s. The small magnitude of the disturbance estimation error indicates that the proposed observer exhibits high disturbance estimation accuracy. Figure 3 presents the attitude angle tracking response, showing that the system is able to accurately track the desired attitude angles. This indicates that our proposed controller can exhibit strong robustness and effectively mitigate the impact of disturbances on attitude tracking. As shown in Figure 4 and Figure 5, the corresponding tracking error signals satisfy the prescribed performance constraints and rapidly converge to a sufficiently small bound within 2 s. This demonstrates that the controller not only ensures tracking accuracy but also meets the corresponding performance requirements. Additionally, as shown in Figure 6, the control input torques required for attitude tracking remain within the physical limitations of the actuators, indicating the practical feasibility of the controller in this paper. In conclusion, the proposed control scheme effectively achieves the desired tracking of the satellite attitude system under the external disturbances, meeting the prescribed tracking performance requirements while exhibiting the fast response and robustness. Then, the above simulations validate both the effectiveness and practical feasibility of the proposed controller.

**Figure 7:**
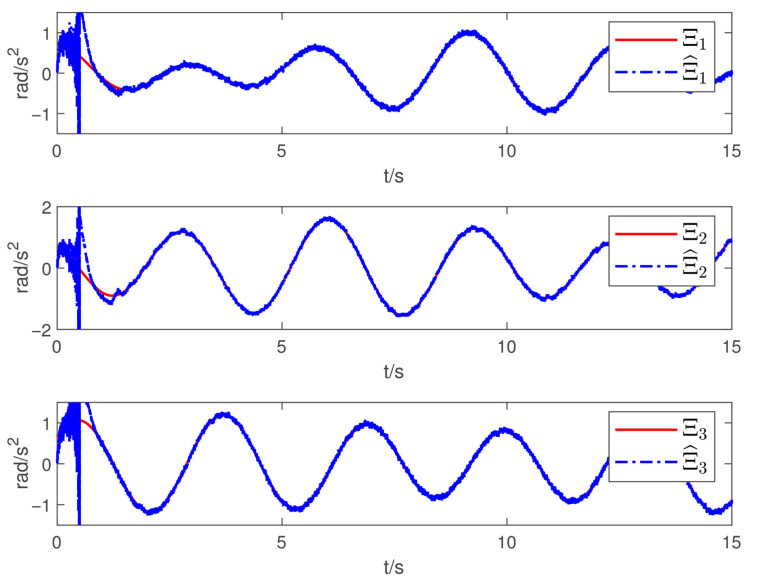
Responses of the disturbance estimation with measurement noise.

**Figure 8:**
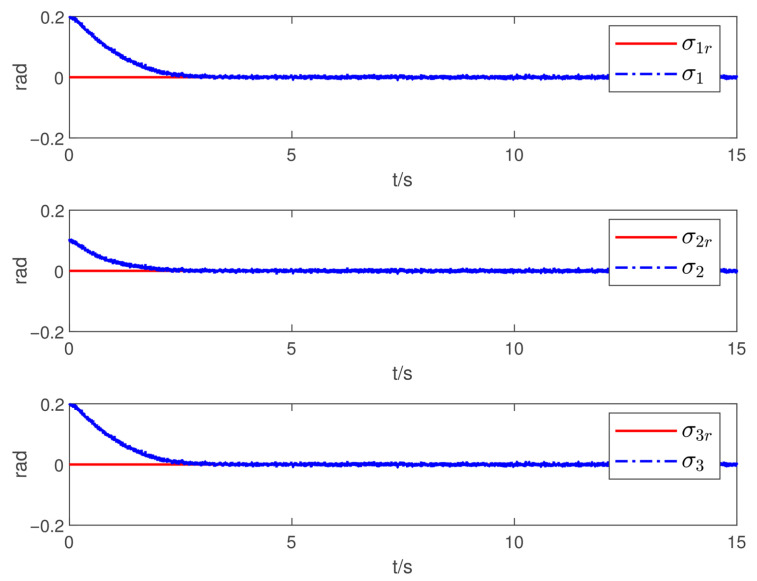
Responses of the attitude-tracking error with measurement noise.

**Figure 9:**
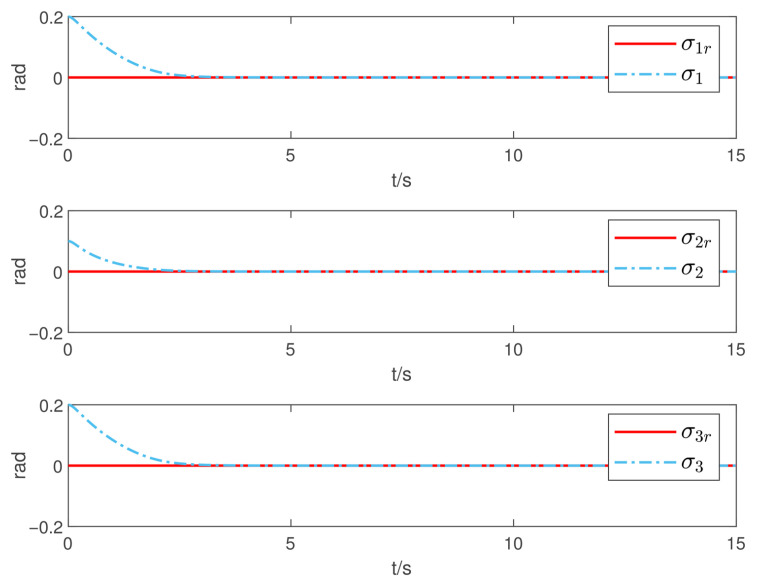
Tracking responses of satellite attitude angle under model uncertainties.

Moreover, in the actual space environments, the satellite attitude system always encounters the measurement noise and model uncertainties, which may unavoidably degrade the control performance. Thus, in what follows, we will respectively consider the effects of two unfavorable factors above, and present corresponding simulations to show the great applicability of our proposed controller. Firstly, the measurement noise is assumed to be Gaussian white noise with zero mean and a variance of $1\times 10^{-5}$ impacting on measured states, namely σ and ω. By using the identical parameters used above, the simulating results of disturbance estimation and attitude-tracking errors are presented in Figure 7 and Figure 8. Though the estimation and tracking performance slightly decrease, the desired control objective still remains achievable despite the influence of measurement noise. Secondly, we introduce model uncertainties into the satellite attitude system, and the system model (1) can be rewritten as

$$\begin{array}{c} \left\{\begin{array}{c} \dot{\sigma }=\frac{1}{4}\left[\left(1-\sigma^{T}\sigma \right)I_{3}+2\sigma^{\times }+2\sigma \sigma^{T}\right]\omega =G\left(\sigma \right)\omega \\ J\dot{\omega }=-\omega^{\times }J\omega +Du+d+\Delta \left(\omega \right) \end{array}\right. \end{array}$$

where the uncertainty Δ(ω) is set as 0.2cos(ω). Then, the simulation results are illustrated in Figure 9, which implies that our controller not only can achieve effective tracking control but also can maintain strong robustness under parameter uncertainties. Therefore, even under the influences of measurement noise and parameter uncertainties, the proposed control scheme can still be applicable in certain complex and practical situations.

## 7. Conclusions

This paper has developed the PPC-based control strategy to ensure the accurate tracking control of rigid satellites subject to external disturbances, while ensuring the prescribed tracking performance. Firstly, the PTDO was introduced to estimate the external disturbances within the prescribed time. Secondly, the PPC-based controller was developed to guarantee that both the transient and steady-state performance of the satellite attitude system satisfied the prescribed requirements. Thirdly, based on Lyapunov stability theory, it was demonstrated that the disturbance estimation error and the unconstrained error of the attitude system are uniformly ultimately bounded, thereby ensuring the desired control performance. Finally, the effectiveness of the proposed control scheme was validated by resorting to simulation experiments.

## Data Availability

The data is available upon request.
